# A Systematic Review of Clinical Pharmacokinetics of Inhaled Antiviral

**DOI:** 10.3390/medicina59040642

**Published:** 2023-03-23

**Authors:** Mohammed Kanan Alshammari, Mohammed Salem Almutairi, Mohammed Dakhilallah Althobaiti, Watin Ahmed Alsawyan, Samar Abdulrahman Alomair, Raghad Rsheed Alwattban, Zahra Hassan Al Khozam, Taif Jundi Alanazi, Abdullah S. Alhuqyal, Hassan Saud Al Darwish, Abdulaziz Faisal Alotaibi, Fahad Naif Almutairi, Abdullah Abdulrahman Alanazi

**Affiliations:** 1 Department of Clinical Pharmacy, King Fahad Medical City, Riyadh 12211, Saudi Arabia; 2 Maternity and Children Hospital, Dammam 32274, Saudi Arabia; 3 Department of Nursing Adult Medical Surgical, Third Health Cluster, Riyadh 17451, Saudi Arabia; 4 Medicine College, Qassim University, Ar Rass 58884, Saudi Arabia; 5 Ministry of Health, Second Health Cluster, Riyadh 11375, Saudi Arabia; 6 Department of Medicine, Qassim University, Ar Rass 51941, Saudi Arabia; 7 Department of Clinical Pharmacy, Faculty of Pharmacy, Northern Border University, Rafha 91911, Saudi Arabia; 8 Department of Medicine, Shaqra University, Riyadh 17451, Saudi Arabia; 9 Department of Medical Collage, Shaqra University, Dawadmi 17451, Saudi Arabia; 10 Directorate of Health Affairs, Ministry of Health, Hafar Al-Batin 39511, Saudi Arabia

**Keywords:** systematic review, clinical use, pharmacokinetics, inhaled, antiviral

## Abstract

*Background and Objectives*: The study of clinical pharmacokinetics of inhaled antivirals is particularly important as it helps one to understand the therapeutic efficacy of these drugs and how best to use them in the treatment of respiratory viral infections such as influenza and the current COVID-19 pandemic. The article presents a systematic review of the available pharmacokinetic data of inhaled antivirals in humans, which could be beneficial for clinicians in adjusting doses for diseased populations. *Materials and Methods*: This systematic review followed the Preferred Reporting Items for Systematic Reviews and Meta-Analyses (PRISMA) 2020 guidelines. A comprehensive literature search was conducted using multiple databases, and studies were screened by two independent reviewers to assess their eligibility. Data were extracted from the eligible studies and assessed for quality using appropriate tools. *Results*: This systematic review evaluated the pharmacokinetic parameters of inhaled antiviral drugs. The review analyzed 17 studies, which included Zanamivir, Laninamivir, and Ribavirin with 901 participants, and found that the non-compartmental approach was used in most studies for the pharmacokinetic analysis. The outcomes of most studies were to assess clinical pharmacokinetic parameters such as the Cmax, AUC, and t1/2 of inhaled antivirals. *Conclusions*: Overall, the studies found that the inhaled antiviral drugs were well tolerated and exhibited favorable pharmacokinetic profiles. The review provides valuable information on the use of these drugs for the treatment of influenza and other viral respiratory infections.

## 1. Introduction

Clinical pharmacokinetics studies are crucial in the context of inhaled antivirals. They clarify the therapeutic efficacy of these medications and the optimal ways to administer them to treat respiratory viral diseases such as influenza and the current COVID-19 [1,2]. Inhaled antivirals refer to a class of drugs that are delivered directly to the respiratory tract through inhalation, as opposed to being taken orally or intravenously [3]. This method of administration offers several advantages over other routes, including a faster onset of action, more direct and localized exposure to the site of infection, and lower systemic drug exposure and associated side effects [4]. Although there are certain advantages to inhaled medication administration, this route also has significant drawbacks, particularly when it comes to achieving accurate and repeatable doses [5]. The amount of medication that is delivered to the lungs and, consequently, the therapeutic effect can be influenced by a number of variables. These include the size of the drug particles, the shape of the inhaler device, patient technique, anatomy of the airway, pathophysiological effects of acute and chronic diseases, and environmental factors [6,7].

The pharmacokinetics of inhaled antivirals can be divided into three main phases: deposition, absorption, and elimination [8]. Deposition refers to the amount of drug that is delivered to the respiratory tract and deposited in the lungs. The optimal drug deposition site after inhalation varies based on the indication and the physical and chemical characteristics of the drug [9]. Absorption refers to the process by which the drug moves from the lungs into the site of action, where it can then exert its therapeutic effect [10]. The rapid absorption of small molecules within alveolar cells can make it challenging to measure plasma drug concentrations after inhalation. This sets the pharmacokinetic study of inhaled antimicrobials apart from traditional pharmacokinetic studies, as it requires a highly sensitive assay and frequent sampling over a short period to accurately determine the absorption rate [11]. The elimination of inhaled antivirals from the body can occur via multiple pathways, including mucociliary clearance, removal via alveolar macrophages, metabolism, excretion, penetration into systemic circulation, and exhalation. The specific pathways used for elimination may vary based on factors such as the specific antiviral, patient characteristics, and the disease state [12]. It is important to consider the pharmacokinetics of inhaled antivirals when determining the dosing regimen for these drugs. The goal is to achieve therapeutic concentrations of the drug in the respiratory tract, while minimizing systemic exposure and the associated side effects [13]. Understanding the interplay between drug delivery, absorption, and elimination is essential for optimizing dosing regimens and ensuring that patients receive the maximum therapeutic benefit from these drugs.

Inhaled antivirals offered several advantages over oral or intravenous formulations of these drugs during the COVID-19 pandemic [14]. First, inhaled drugs reach the site of infection more quickly, leading to a faster onset of action and improved therapeutic outcomes. Second, inhaled drugs are delivered directly to the respiratory tract, which minimizes systemic exposure and reduces the risk of side effects that may be associated with oral or intravenous formulations of these drugs [15]. In addition, inhaled antivirals have been shown to be effective in reducing the severity and duration of respiratory viral infections, as well as reducing the risk of hospitalization and death in some cases [16]. This is particularly important for individuals who are at high risk of complications from these infections, such as the elderly, young children, and individuals with underlying medical conditions [17]. Dry powder inhalers (DPIs), including the Diskhaler and Rotahaler, are significant drug delivery devices for inhaled antiviral drugs such as Zanamivir, Laninamivir, and Ribavirin. DPIs are particularly useful for delivering drugs to the lungs, where respiratory viral infections are most prevalent.

The main objective of this systematic review was to fill the gap in knowledge regarding the pharmacokinetics of inhaled antiviral drugs. While there is a significant amount of literature on the pharmacokinetics of systemically administered antivirals, there is a lack of comprehensive reviews on the pharmacokinetics of inhaled antivirals. The authors therefore aimed to systematically review the existing literature to provide a comprehensive overview of the pharmacokinetic aspects of inhaled antivirals in humans. To date, no systematic review has been conducted to determine the PK parameters of inhaled antivirals. This review endeavors to systematically gather, summarize, and analyze all of the available PK data of inhaled antivirals in the human population, which could be beneficial for clinicians in adjusting the doses in both healthy and diseased populations.

## 2. Materials and Methods

The methodology used for conducting this systematic review on the pharmacokinetics of inhaled antivirals in humans was the Preferred Reporting Items for Systematic Reviews and Meta-Analyses (PRISMA) guidelines.

### 2.1. Identification of the Research Question

The research question was identified, which was clear, specific, and focused on the pharmacokinetics of inhaled antivirals in humans following the inhalation route of administration.

### 2.2. Literature Search

A comprehensive literature search was conducted using multiple databases, such as PubMed, Google Scholar, Science Direct, Embase, and the Cochrane Library, to identify relevant studies. The search was performed using relevant keywords and Medical Subject Headings (MeSH) terms. These databases were searched with the following key terms: ‘Inhaled antivirals’, ‘antivirals’, ‘Inhaled antimicrobial’, ‘Inhaled antibiotic’, ‘Respiratory Infections’, ‘clinical pharmacokinetic’, and ‘pharmacokinetic’ with “AND” or “OR”. An overview of the final search strategy with MeSH terms and text words for each of the four domains is described in Table 1.

### 2.3. Inclusion and Exclusion Criteria

The inclusion criteria were clearly defined and included studies that examined the PK parameters of inhaled antivirals in humans following inhalation. The studies were limited to those conducted in healthy and diseased populations. Only peer-reviewed articles in the English language and at least one reported PK parameter that specified an inhaled antiviral were included. Both randomized control trials (RCTs) and observational studies were included. Moreover, there was no limitation on the publication year, whereas the studies that did not meet the inclusion criteria or those that did not provide PK data of inhaled antivirals were excluded.

### 2.4. Study Selection

The studies identified through the literature search were screened by two independent reviewers to assess their eligibility. Any disagreements were resolved through consensus or by a third reviewer. After evaluating all of the databases, studies were screened for duplication detection, which were then deleted, respectively. The articles were excluded after screening title and abstract. Review articles and book chapters were also excluded.

### 2.5. Data Extraction

Data were extracted from the eligible studies using a standardized data extraction form. The data included information on study reference, design, research objectives, outcome measures, spray device, model structure, population characteristics, sample size, drug name, dosing practice, and pharmacokinetic parameters, including plasma concentration time curve (AUC), peak concentration of antivirals in plasma (Cmax), half-life (T1/2), volume of distribution (Vd), and clearance (Cl). The author’s name with publication year and the number were also mentioned.

### 2.6. Quality Assessment

The quality of the eligible studies was assessed using appropriate tools, such as the Cochrane Risk of Bias tool for randomized controlled trials and the Newcastle–Ottawa Scale (NOS) for cohort studies.

### 2.7. Data Synthesis and Reporting

The extracted data were synthesized and analyzed to provide a comprehensive overview of the PK aspects of inhaled antivirals in humans following inhalation.

## 3. Results

### 3.1. Literature Search

**Figure 1:**
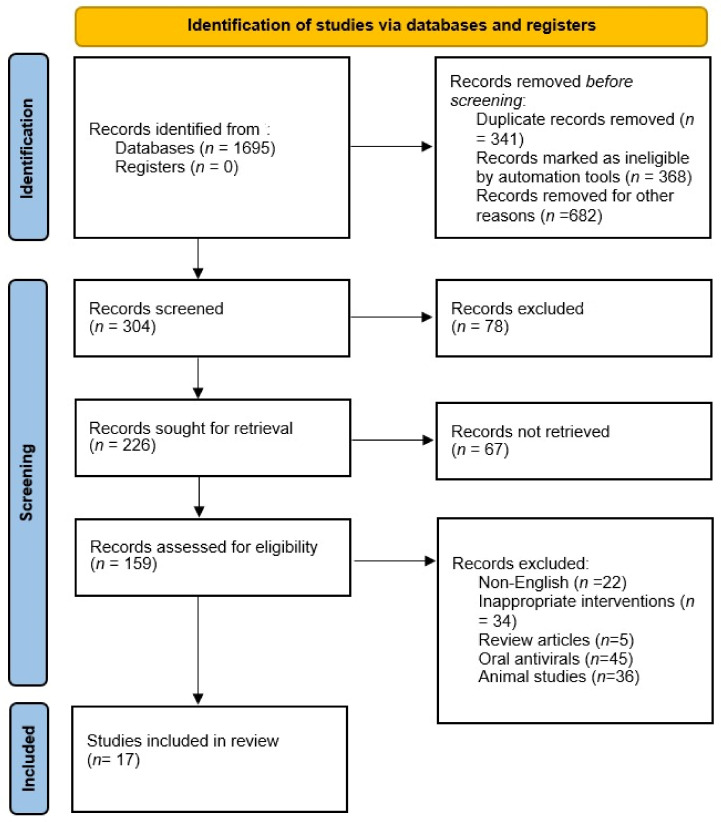
Flow chart of included studies.

A total of 1102 relevant published articles were identified from the gray literature and databases such as PubMED, ScienceDirect, Web of Science, and EMBASE. Of 1695 articles, 341 articles were screened after the exclusion of duplicates. On the basis of the exclusion and inclusion criteria, 1050 articles were excluded after the screening of the titles and abstracts of the articles. Seventy-eight of the included articles that did not contain any information regarding the pharmacokinetics of inhaled antiviral agents were excluded. After the screening of abstracts and full-text articles, 142 articles were excluded for the following reasons: non-English (N = 22), articles on other modes of administration (N = 45), inappropriate interventions (N = 34), review articles (N = 5), and animal studies (N = 36). Data extraction was performed for 17 full-text articles with data on the PKs of inhaled antivirals (Figure 1).

### 3.2. Characteristics of Studies

Seventeen articles met the inclusion criteria for this systematic review [8,10,13,18,19,20,21,22,23,24,25,26,27,28,29,30,31]. The characteristics of selected articles are listed in Table 2. The majority of the articles studied the pharmacokinetic parameters of Zanamivir and Laninamivir. Of 17 articles, 11 studies were randomized controlled trials (RCTs) [8,13,18,19,20,21,22,23,24,25,26], followed by preliminary studies [27,28], open-label studies [10,29], prospective studies [30], and non-randomized studies [31]. The non-compartmental approach was used in 12 studies for the pharmacokinetic analysis. The outcomes of most of the studies were to assess the clinical pharmacokinetics parameters such as maximum concentration (Cmax), area under curve (AUC), and half-life (t1/2) of inhaled antivirals. A total of 901 patients participated in the studies. The majority of the studies used a Rotahaler and Diskhaler for the administration of the drug [13,19,25,30,31].

### 3.3. Quality Assessment of the Studies

The quality of the studies was assessed and summarized in the Supplementary File (Tables S1 and S2). On the basis of the NOS, five articles were rated as 7 and one article scored 6. Overall, the quality score for prospective and preliminary studies was seven. For RCT, the Cochrane bias tool assessed that most of the domains were at low risk.

Pharmacokinetic parameters of inhaled antiviral drugs.

### 3.4. Zanamivir

In most of the studies, the Zanamivir was administered via Diskhaler (Table 3) [13,19,30,31]. Doses utilized in the studies ranged from 4 mg to 16 mg and were administered in either a single dose or multiple doses. However, Weller and his colleagues support the use of Rotahaler/Rotacap especially in an influenza pandemic [13]. The clearance of this drug was 49 L/h via Rotahaler while the clearance was 54 L/h via Diskhaler. Overall, the half-life ranged from 2–3 h when administered via Diskhaler [13,19,30]. In another study, single doses of 4, 8, and 16 mg and multiple doses of 16 mg BID on Day 1 followed by QID for 6 days of Zanamivir were well tolerated when administered via nebulizer and dry powder inhaler [18].

A dose of 10 mg of Zanamivir was well tolerated and safe in pediatric patients [8,30]. Zenamivir was administered twice daily for 5 days intranasally and via being inhaled orally, and the maximum concentration was achieved by 1.5 h after dosing [30]. However, another study reported that the systemic absorption of Zanamivir following oral inhalation or intranasal administration was low [8].

### 3.5. Laninamivir

The majority of the studies reported that the dose of 40 mg of prodrug CS-8958 was well tolerated and exhibited a PK profile, suggesting the potential for the parameters of Laninamivir and its prodrug CS-8958 in healthy participants and patients with comorbidities (Table 3) [20,21]. The maximum concentration of CS-8958 ranged from 12.8 to 433 ng/mL. Ishizuka and her colleagues reported that CS-8958 was well tolerated in patients with renal impairment [20]. The PK parameters such as AUC0-inf, Cmax, and time to Cmax of CS-8958 did not change with the degree of renal impairment; however, the t1/2 of CS-8958 gradually increased with increased renal insufficiency [20].

A study conducted in adults and pediatric patients reported that the volume of distribution of Laninamivir Octanoate (LO) and metabolic clearance of LO were altered with body weight [23]. For a single inhaled dose of 40 mg of LO, the Laninamivir amount was evaluated to be approximately 0.46 mg in the respiratory tract compartment at 1-week post-dose [29].

The concentrations of Laninamivir in the epithelial lining fluid (ELF) and bronchoalveolar lavage (BAL) fluid samples were assessed by Ishizuka and her colleagues to study the drug distribution in airways [10]. The ELF concentration profiles of Laninamivir showed the potential long-lasting effect for the treatment of patients with influenza virus infection [10].

### 3.6. Ribavirin

The studies reported that the utilization of aerosolized Ribavirin was well tolerated in healthy volunteers and the patient with other conditions (Table 3) [24,25,27,28]. Dumont and his colleagues developed dry powder particles using PRINT technology. The doses of 60 mg and 120 mg followed by 30 mg twice daily for 14 days were administered via Rotahaler to healthy volunteers and to those with chronic obstructive pulmonary disease (COPD). They concluded that PRINT formulation was an efficient and convenient mode of administration of the drug to the lungs while minimizing systemic exposure [25]. Moreover, a randomized, placebo-controlled study was performed to evaluate the safety and pharmacokinetics of inhaled Ribavirin [24]. The participants were recruited in four groups where they received different doses. Cohort 1 received 50 mg/mL Ribavirin/placebo (10 mL total volume); Cohort 2 received 50 mg/mL Ribavirin/placebo (20 mL total volume); Cohort 3 received 100 mg/mL Ribavirin/placebo (10 mL total volume); and Cohort 4 received 100 mg/mL Ribavirin/placebo (20 mL total volume). The mean maximum observed concentration (Cmax) and area under the curve (AUC) values were higher in Cohort 4, whereas Cohorts 2 and 3 showed similar PK values. The data support the development of Ribavirin as an empirical treatment option in patients with coronavirus.

In pediatric patients with suspected respiratory syncytial virus infection, patients received aerosolized Ribavirin of 60 mg/mL for 2 h periods TID for 3 days [27]. After the first dose, the mean peak Ribavirin level ranged from 1725 to 2179 mol/L in secretions and 3.8 mol/L in plasma. Ribavirin was rapidly cleared with a mean t1/2 of 1.9 h.

### 3.7. Rimantadine

The safety and pharmacokinetics of rimantadine were assessed by administering it via small-particle aerosol and oral inhalation in healthy volunteers and volunteers with acute influenza virus infection [26]. Rimantadine was delivered at a concentration of 20 µg/L every 4–12 h and 40 µg/L every 15 min to 4 h of air. The clearance of rimantadine ranged from 25.3 to 29.9 L/h and Vd ranged from 904 to 906 L. Some of the participants experienced nasal burning and irritation. This study concluded that a concentration of 20 µg/liter of air was well tolerated for up to 12 h by normal volunteers as well as in those with acute influenza virus infection.

## 4. Discussion

In this systematic review, we have investigated the pharmacokinetic parameters of inhaled antivirals. The non-compartmental model was used in the majority of the studies. Non-compartmental modeling is commonly used to study the pharmacokinetic (PK) parameters of drugs because it provides a simple and efficient way to analyze drug concentration–time data without making any assumptions about the underlying biological system [32]. This makes it particularly useful when the pharmacokinetics of a drug are not well understood or when the data are limited. The studies utilized dry powder inhalers (DPI) such as Diskhaler and Rotahaler for drug delivery. DPIs are used to deliver the drug directly to the lungs and are preferred for different types of drugs and inspiratory flow rates [33]. They are easy to use and have a lower environmental impact compared to other types of inhalers. Overall, drug powder inhalers can have a significant impact on the pharmacokinetic parameters of a drug, which can affect its overall effectiveness and safety. It is important to carefully consider the particle size and density of the drug powder when designing inhaler devices to ensure that medications are delivered effectively and efficiently to the lungs.

While emerging respiratory infectious diseases and associated morbidity and mortality have abated, they remain substantial threats to the public as well as scientific/medical communities [34,35]. Among highly contagious respiratory infections, influenza is responsible for significant morbidity and mortality worldwide [36]. Neuraminidase inhibitors such as oseltamivir, Zanamivir, and Laninamivir have been the mainstay of influenza antiviral treatment over the past few decades [37,38]. Zanamivir is a widely used drug as a therapeutic and prophylactic and is currently available in dry powder inhalation and IV formulation. The recommended dose for prophylaxis is 10 mg once daily for up to 28 days in adults and for pediatric patients aged above 5 years treatment is 10 mg twice daily for 5 days [39]. In a study, it was reported that about 12% of the dose is absorbed systemically after inhaled administration and reached Cmax within 2 h [19]. Approximately 78% and 13% of the drug Zanamivir was deposited in the oropharynx and the lungs when administered intranasally [18,40]. In these studies, Zanamivir was considered to be a well-tolerated antiviral drug administered intranasally by healthy volunteers and patients with influenza.

Furthermore, Laninamivir, a substituted compound of Zanamivir, is a therapeutic agent for the prophylaxis and treatment of viral infections [41,42]. Among the prodrugs of Laninamivir, Laninamivir Octanoate (LO), also known as CS-8958, is the most potent drug for the treatment of influenza available in only orally inhaled formulation, which delivers the drug directly to the respiratory tract [40]. Multisite studies have reported that LO has prophylactic as well as therapeutic efficacy against highly pathogenic H5N1 influenza viruses [43,44]. A single inhaled dose of 20 mg in pediatric patients <10 years old or 40 mg in adults and children >10 years old of LO was found to be more effective and safe over oseltamivir regimens concerning mean time to illness alleviation [45]. The pharmacokinetic profile of LO has been assessed in healthy adults, patients with renal insufficiency, elderly subjects, and patients with influenza infection [20,21,23]. Linear PKs of Laninamivir and its prodrug was observed across a wide range of doses (5–120 mg) by Yoshihara et al. [23]. The peak of plasma concentration was achieved after dosing and then declined with a half-life of approximately 2 h, whereas the peak of plasma Laninamivir concentration was achieved at approximately 4 h post-dose and declined with a half-life of approximately 3 days [21,23]. The findings documented that the single inhaled dose of LO was sufficient to treat influenza virus infection; moreover, it may show better compliance in patients with influenza infection and this feature may be favorable for prophylactic use.

Besides influenza virus infection, respiratory syncytial virus (RSV) is the most important cause of serious lower respiratory tract infections (LRTIs), especially in pediatric patients [46,47]. Ribavirin, an inosine monophosphate dehydrogenase inhibitor, is currently recommended for the treatment of LRTIs in hospitalized patients with RSV [48]. Aerosolized Ribavirin has shown significant clinical improvement in patients with RSV [49,50]. The PK results are promising, specifically in the context of coronavirus and intensive care settings where patients’ ability to swallow is compromised, and the delivery of a drug to the site of infection (respiratory tract) provides advantages over oral and intravenous formulations [51]. Ribavirin exhibits two rapid phases, i.e., absorption and distribution and a long terminal clearance phase [52,53].

Aerosolization provides the direct delivery of antiviral drugs to the site via the respiratory tract in a desirable amount to remove pathogenic organisms [54]. This method of drug administration usually results in a higher concentration of the drug at the site of infection than systematic administration does, and may minimize systemic toxicities [55]. Rimantadine has better antiviral activity against influenza A virus strains than amantadine. Hayden et al. have previously reported that the use of rimantadine delivered via an ultrasonic nebulizer in subjects with influenza A virus is well tolerated except for minor complaints, i.e., unpleasant smell or taste [56]. However, Atmar et al. reported that nasal burning or irritation was the most common side effect associated with rimantadine SPA [26]. About 45.6% of the dose of rimantadine reached the systemic circulation after SPA administration, and the mean peak levels in serum were 8.6-fold lower [57]. The studies found that Ribavirin was rapidly cleared, with a t1/2 of 1.9 h. SPA delivery of antiviral drugs has been shown to be effective in the treatment of other respiratory viral infections [26,58].

The novelty of this systematic review lies in its comprehensive and systematic approach to synthesizing the available literature on the clinical pharmacokinetics of inhaled antiviral drugs. The review provides a detailed and up-to-date summary of the pharmacokinetic parameters of various inhaled antiviral drugs, which can help to inform clinical decision-making and optimize the use of these drugs in the treatment of respiratory viral infections. Additionally, the review highlights the gaps in current knowledge and identifies areas for further research, which can guide future studies in this field. Overall, this systematic review tried to provide details of the inhaled antiviral agents, and offered a few inhaled antiviral drugs for the treatment of respiratory viral infections. A limited amount of data on the clinical pharmacokinetics of inhaled antiviral drugs is still a matter of concern. Several factors such as inspiratory flow rate, tidal volume, and presence and degree of airway obstruction can affect the concentration of antimicrobials [40]. However, the data on these factors are limited. Few studies are performed during the preclinical and clinical stages. It is hereby recommended that more studies are required for the further development of a novel aerosolized drug.

## 5. Conclusions

The clinical pharmacokinetics (PKs) of inhaled antivirals have been studied using non-compartmental models. Despite the aforementioned factors, clinical PK studies of inhaled antivirals have shown that they result in high concentrations in the respiratory tract, with relatively low systemic exposure, and reduce the risk of toxicity, which leads to the development of such advanced formulations and aids in modifying advanced aerosolization devices. Moreover, limited data were available on the pharmacokinetic parameters of existing inhaled drugs. Therefore, further studies, especially randomized controlled trials, are required to obtain a PK profile of inhaled antiviral drugs.

## Figures and Tables

**Table 1:** Overview of final search strategy with MeSH terms and text words for each of the four domains (pharmacokinetics, antivirals, characteristics of patient, and type of population).

Pharmacokinetics	Antivirals	Characteristics of Patient	Type of Population
MeSH terms	MeSH terms	MeSH terms	MeSH terms
Clinical pharmacokinetics (MeSH) Clinical pharmacokinetics (subheading) Drug monitoring (subheading) Pharmacokinetic analysis (subheading)	Antiviral agents (MeSH) Antimicrobial agents (MeSH) Inhaled antiviral agents (MeSH)	Healthy volunteers Patients with viral infection Critically ill	Pediatric patients (MeSH) Adolescents (MeSH) Adults (MeSH)
Title/Abstract	Title/Abstract	Title/Abstract	Title/Abstract
Pharmacokinetic, pharmacodynamic, target attainment, area under curve, maximum plasma concentration, drug monitoring, drug deposition	See electronic and supplementary file	Influenza, healthy, subjects, volunteers, renal impairment, critically ill, ICU patients, PICU	Children, workers, youth, males, females, elderly, adults, babies

Keywords within each domain were combined with OR; all domains were combined with AND, as shown in Supplementary File.

**Table 2:** Main characteristics of studies on inhaled antiviral drugs.

Study	Research Objectives	Study Design	Outcome Measures	Sample Size	Spray Device	Model Structure
Zenamivir						
Cass et al., 1999 [18]	To assess the clinical PKs and safety of Zanamivir.	RCT	C_max_, AUC, t_1/2_, Cl, V_d_	104	Nebulizer/dry powder inhaler	Non-compartmental models
Weller et al., 2013 [13]	To evaluate preliminary PKs and safety data to support the use of Zanamivir via Rotahaler/Rotacap.	RCT	C_max_, AUC, T_max_, t_1/2_	18	Rotadisk/Diskhaler	Mixed-effect models
Cass et al., 1999 [19]	To determine the sites of Zanamivir deposition in the respiratory tract and the PKs of Zanamivir via Diskhaler device and a prototype device.	RCT	C_max_, AUC, t_1/2_, Cl	13	Diskhaler/prototype device	Non-compartmental models
Peng et al., 2000 [8]	To assess the population PKs of Zenamivir in participants with experimental and naturally occurring influenza.	RCT	Cl, V_d_	201	Intranasal and inhaled powder	Mixed-effect models
Peng et al., 2000 [30]	To examine the PKs, safety, and tolerability of Zanamivir in pediatric patients.	Prospective study	C_max_, AUC, T_max_, t_1/2_, V_d_	18	Nebulizer and Diskhaler	Non-compartment model
Shelton et al., 2011 [31]	To evaluate serum as well as pulmonary PKs following IV and oral inhaled administration.	Non-randomized study	C_max_, AUC, T_max_, t_1/2_, Cl, V_d_	42	Diskhaler and Rotahaler	Non-compartmental model
Laninamivir						
Ishizuka et al., 2011 [20]	To assess the safety and PKs of Laninamivir after administration of its prodrug, CS-8958.	RCT	C_max_, AUC, T_max_, t_1/2_, V_d_	20	Flowcaps dry powder inhaler	Non-compartment model
Ishizuka et al., 2010 [21]	To evaluate its safety, tolerability, and PKs after inhaled administration of its prodrug, CS-8958.	RCT	C_max_, AUC, T_max_, t_1/2_, Cl, V_d_	76	Flowcaps dry powder inhaler	Non-compartmental model
Yoshiba et al., 2011 [22]	To measure the PK parameters of Laninamivir using a new easy-to-use inhaler.	RCT	C_max_, AUC, T_max_, t_1/2,_ Cl, V_d_	16	Flow powder inhaler	Non-compartmental model
Ishizuka et al., 2012 [10]	To determine the intrapulmonary PKs of LO and Laninamivir.	Open-label study	C_max_, AUC, T _max_, T_1/2_	36	Dry powder inhaler	Non-compartmental model
Toyama et al., 2017 [29]	To evaluate the safety and PKs of Nebulized Laninamivir Octanoate.	Open-label study	T_1/2_	40	Reusable nebulizer	Non-compartmental model
Yoshihara et al., 2013 [23]	To study the population PKs of LO and Laninamivir in healthy subjects, and adult and pediatric patients with influenza virus infection from eight clinical studies, and to evaluate covariate effects on PKs.	RCT	C_max_, AUC, T_1/2_, Cl	175	Prototype and commercial	One- and two-compartmental models
Ribavirin						
Couroux et al., 2022 [24]	To assess the safety and PKs of four, single-dose regimens of Ribavirin aerosol.	RCT	C_max_, AUC, t_1/2,_	32	Air-jet nebulizer	Non-compartmental model
Englund et al., 1990 [27]	To evaluate the safety of high-dose, short-duration Ribavirin aerosol therapy in pediatric patients with suspected RSV infection. To evaluate the drug concentrations in blood and respiratory secretions and antiviral effects.	Preliminary study	C_max_, t_1/2_	9	Aerosol nebulizer	Not stated
Linn et al., 1995 [28]	To evaluate exposure variables including aerosol concentration, duration of exposure, aerosol size range, and exposed participants’ ventilation rates.	Preliminary study	C_max_, t_1/2_	14	Aerosol nebulizer	Not stated
Dumont et al., 2020 [25]	To assess the efficient delivery of Ribavirin using the particle replication in non-wetting templates (PRINT) technology.	RCT	C_max_, AUC, T_max_	60	Rotahaler	Non-compartmental model
Rimantadine						
Atmar et al., 1990 [26]	To assess the safety and PKs of rimantadine in healthy adults and adults with acute influenza virus infection.	RCT	AUC, t_1/2_, Cl, V	27	Collison nebulizer	Non-compartmental model

RCT = randomized controlled trial, C_max_ = maximum concentration, AUC = area under curve, T_1/2_ = half-life, V_d_ = volume of distribution, Cl = clearance, PKs = pharmacokinetics, LO = Laninamivir Octanoate, T_max_ = time to peak drug concentration.

**Table 3:** Pharmacokinetic parameters of inhaled antiviral drugs.

Drug/Study	Dosing Practice	Pharmacokinetic Parameters				
		C_max_	T_1/2_	AUC	V_d_	Cl
Zenamivir						
Cass et al., 1999 [18]	Single and multiple doses of 8 and 16 mg six times daily for 5 days	**Nebulizer:** 63–139 µg/L **Dry powder inhaler:** Cmax: 39–54 µg/L	**Nebulizer:** 2.21 h **Dry powder inhaler:** 3.56 h	**Nebulizer** 425 µg.h/L **Dry powder inhaler** 160 µg.h/L	Not stated	Not stated
Weller et al., 2013 [13]	A dose of 10 mg via oral inhalation every 12 h for 5 days using Rotadisk/Diskhaler A dose of 10 mg via oral inhalation every 12 h for 5 days using Rotacap/Rotahaler	**Diskhaler:** 32 ng/mL **Rotahaler:** 37 ng/mL	**Diskhaler:** 3 h **Rotahaler:** 3.2 h	**Diskhaler:** 133 ng.h/mL **Rotahaler:** 157 ng.h/mL	Not stated	**Diskhaler:** 54 L/h **Rotahaler:** 49 L/h
Cass et al., 1999 [19]	A dose of 10 mg using Diskhaler A dose of 10 mg using prototype device	**Diskhaler:** 34 µg/L **Prototype device:** 30 µg/L	**Diskhaler:** 2.5 h **Prototype device:** 3.0 h	**Diskhaler** 184 µg.h/L **Rotahaler** 190 µg.h/L	Not stated	**Diskhaler** 54.3 L/h **Rotahaler** 52.6 L/h
Peng et al., 2000 [8]	Placebo/Zanamivir twice daily for 5 days either intranasally (10 mg) or orally inhaled (6.4 mg)	Not stated	Not stated	Not stated	**Intranasal:** 296 L **Inhaled powder:** 161 L	**Intranasal:** Cl: 74.1 L/h **Inhaled powder:** Cl: 40 L/h
Peng et al., 2000 [30]	A dose of 10 mg inhaled by nebulizer A dose of 10 mg inhaled by Diskhaler	**Nebulizer:** 47 µg/L **Diskhaler:** 40–47 µg/L	**Nebulizer:** 1.9 h **Diskhaler:** 2 h	**Nebulizer:** 184 µg. h/L **Diskhaler:** 167–192 µg.h/L	**Nebulizer:** 16.9 L **Diskhaler:** 3.5–6.9 L	**Nebulizer:** 54.3 L/h **Diskhaler:** 52.1–60 L/h
Shelton et al., 2011 [31]	10 mg q12h for two doses	21.2 ng/mL	1.75 h	175 ng.h/mL	Not stated	Not stated
Laninamivir						
Ishizuka et al., 2011 [20]	A single inhaled dose of 20 mg	**CS-8958:** 57.4–74.3 ng/mL **Laninamivir:** 14.5–29.9 ng/mL	**CS-8958:** 2.3–5.3 h **Laninamivir:** 53.2–57 h	**CS-8958:** 291–401 **Laninamivir:** 426–2223 ng.h/mL	**CS-8958:** 6.5–26 mL/min **Laninamivir:** 12.7–65 mL/min	Not stated
Ishizuka et al., 2010 [21]	Doses of CS-8958 of 5 mg–120 mg	**CS-8958:** 12.8–433 ng/mL **Laninamivir:** 2.6–66 ng/mL	**CS-8958:** 1.7–10.7 h **Laninamivir:** 5.7–80.8 h	**CS-8958:** 45.3–1567 **Laninamivir:** 19.9–2059	**CS-8958:** 193–956 L **Laninamivir:** Not stated	**CS-8958:** 35–61 mL/min **Laninamivir:** 90–576 mL/min
Yoshiba et al., 2011 [22]	Doses of 20 mg and 40 mg	**Laninamivir:** 19–38.3 ng/mL **LO:** 145–336 ng/mL	**Laninamivir:** 66.6–74.4 h **LO:** 1.79–2.70 h	**Laninamivir:** 558–1080 ng.h/mL **LO:** 440–1018 ng.h/mL	**Laninamivir:** Not stated **LO:** 121–160 L	**Laninamivir:** 84.9–106.9 mL/min **LO:** 34.3–35.9 mL/min
Ishizuka et al., 2012 [10]	A single inhaled dose of 40 mg of LO	**Laninamivir:** 0.025–152.3 µg/mL **LO:** 0.162–2085 µg/mL	**Laninamivir:** 45.7 h **LO:** 2.6–89.9 h	**Laninamivir:** 0.826–17,271 µg/mL **LO:** 0.705–31942	Not stated	Not stated
Toyama et al., 2017 [29]	A single dose of 40~320 mg of LO was inhaled	Not stated	**Laninamivir:** 58.3–165.8 h **LO:** 1.8–55.1 h	Not stated	Not stated	Not stated
Yoshihara et al., 2013 [23]	**Group 1:** 5, 10, 20, 40 mg (single dose) **Group 2:** 20, 40 mg (BID, 3 days) **Group 3:** 40 mg (single dose: young and elderly); 20 mg (QID, 2 days: elderly only) **Group 4:** 20 mg (single dose) **Group 5:** 80, 120 mg (single dose) **Group 6** 40 mg (single dose) **Group 7:** 20, 40 mg (single dose); 20 mg (QID, 2 days) **Group 8:** 40 mg (single dose)	Not stated	**Laninamivir:** 4 h **LO:** 2 h	Not stated	Not stated	**Laninamivir:** 5.21 h/L **LO:** 64.8 h/L
Ribavirin						
Couroux et al., 2022 [24]	**Cohort 1:** 50 mg/mL Ribavirin/placebo (10 mL total volume) **Cohort 2:** 50 mg/mL Ribavirin/placebo (20 mL total volume) **Cohort 3:** 100 mg/mL Ribavirin/placebo (10 mL total volume) **Cohort 4:** 100 mg/mL Ribavirin/placebo (20 mL total volume)	**Cohort 1:** 0.63 µg/mL **Cohort 2:** 1.07 µg/mL **Cohort 3:** 0.95 µg/mL **Cohort 4:** 1.64 µg/mL	**Cohort 1:** 1 h **Cohort 2:** 1.75 h **Cohort 3:** 1.5 h **Cohort 4:** 2.0 h	**Cohort 1:** 4.90 µg/mL **Cohort 2:** 10.55 µg/mL **Cohort 3:** 9.27 µg/mL **Cohort 4:** 15.55 µg/mL	Not stated	Not stated
Englund et al., 1990 [27]	A dose of 60 mg/mL for 2 h periods TID for up to 5 days	1725–2179 pmol/L	1.9 h	Not stated	Not stated	Not stated
Linn et al., 1995 [28]	**High exposure group** A dose of 30 mg QID for 4 days **Low exposure group** A dose of 3 mg QID for 4 days	0.89 pmol/L	37–39 h	Not stated	Not stated	Not stated
Dumont et al., 2020 [25]	**Cohort A** Single dose of 60 mg and 120 mg followed by 30 mg BID for 14 days **Cohort B** 60 mg BID for 14 consecutive days	**Cohort A:** 143–508 ng/mL **Cohort B:** 189–285 ng/mL	**Cohort A:** 0.5–0.625 h **Cohort B:** 0.5–0.633 h	**Cohort A:** 578–1490 ng.h/mL **Cohort B:** 565–2060 ng.h/mL	Not stated	Not stated
Rimantidine						
Atmar et al., 1990 [26]	**Rimantadine in water** 40 µg/L of air every 15 min to 4 h **Rimantadine in PBS** 40 µg/L of air after every 4 h **Rimantadine in water** 20 µg/L of air every 4 h to 12 h	Not stated	**Oral:** 25.2 h **SPA:** 24.1 h	**Oral:** 8193 ng.h/mL **SPA:** 1208 ng.h/mL	**Oral:** 904 L **SPA:** 906 L	**Oral:** 25.3 **SPA:** 29.9

C_max_ = maximum concentration, AUC = area under curve, T_1/2_ = half-life, V_d_ = volume of distribution, Cl = clearance, PKs = pharmacokinetics, LO = Laninamivir Octanoate, T_max_ = time to peak drug concentration, BID = two times a day, TID = three times a day, QID = four times a day, PBS = Phosphate buffer solution, SPA = small-particle aerosol.

## Data Availability

Not applicable.

## Supplementary Materials

The following supporting information can be downloaded at https://www.mdpi.com/article/10.3390/medicina59040642/s1, Table S1: Quality assessment of cohort studies; Table S2: Risk of bias assessment for randomized controlled trials.
