# Supplementary material for: A Systematic Review of Clinical Pharmacokinetics of Inhaled Antiviral
Source: Medicina (Kaunas). 2023 Mar 23;59(4):642. doi: 10.3390/medicina59040642 (PMC10145512; doi:10.3390/medicina59040642)
Supplement: Supplementary file 1 [file medicina-59-00642-s001.zip › medicina-2288681-supplementary.pdf]

Table S1. Quality assessment of cohort studies.

Reference	Selection				Comparability	Outcomes		
	Representative of Exposed Studies A	Selection of Non-Exposed B	Ascertainment of Exposure C	Demonstration of Outcome D	Comparability of Cohort Studies on Basis of Design E	Assessment of Outcomes F	Adequacy of Follow-Up G	Quality Score
Peng et al., 2000 [1]	*	*	*	*	*	*	*	7
Shelton et al., 2011 [2]	*	*	*	*	*	*	*	7
Ishizuka et al., 2012 [3]	*	*	*	*	*	*	*	7
Toyama et al., 2017 [4]	*	*	*	*	*	-	*	6
Englund et al., 1990 [5]	*	*	*	*	*	*	*	7
Linn et al., 1995 [6]	*	*	*	*	*	*	*	7

A: *=truly representative or somewhat representative of average in target population. B: *=Drawn from the same community. C: *=Secured record or structured review. D: *=Yes, - = No. E: *= Study controls for age, gender, and other factors. F: *=Record linkage or blind assessment, **=Both. G: *=follow-up of all subjects.

Table S2. Risk of bias assessment for randomized controlled trials.

Study	Random Sequence Generation	Allocation Concealment	Blinding of Participants and Personnel	Blinding of Outcome Assessment	Incomplete Outcome Data	Selective Reporting	Other Bias
Cass et al., 1999 [7]	Low risk	Low risk	Unclear	Low risk	Low risk	Low risk	Unclear
Weller et al., 2013 [8]	Low risk	Low risk	High risk	High risk	Low risk	Low risk	Unclear
Cass et al., 1999 [9]	Low risk	Low risk	Unclear	Low risk	Low risk	Low risk	Unclear
Peng et al., 2000 [10]	Low risk	Low risk	Low risk	Low risk	Low risk	Low risk	Unclear
Ishizuka et al., 2011 [11]	Low risk	Low risk	Low risk	Low risk	Low risk	Low risk	Unclear
Ishizuka et al.,2010 [12]	Low risk	Low risk	Low risk	Low risk	Low risk	Low risk	Unclear
Yoshiba et al., 2011 [13]	Low risk	Low risk	Unclear	Unclear	Low risk	Low risk	Unclear
Yoshihara et al., 2013 [14]	Low risk	Low risk	Low risk	Low risk	Low risk	Low risk	Unclear
Couroux et al., 2022 [15]	Low risk	Low risk	Low risk	Low risk	Low risk	Low risk	Unclear
Dumont et al., 2020 [16]	Low risk	Low risk	Low risk	Low risk	Unclear	Low risk	Unclear
Atmar et al., 1990 [17]	Low risk	Low risk	High risk	High risk	Low risk	Low risk	Unclear

References

1. Peng, A.W.; Hussey, E.K.; Rosolowski, B.; Blumer, J.L. Pharmacokinetics and tolerability of a single inhaled dose of zanamivir in children. *Curr. Ther. Res.* **2000**, *61*, 36–46.
2. Shelton, M.J.; Lovern, M.; Ng-Cashin, J.; Jones, L.; Gould, E.; Gauvin, J.; Rodvold, K.A. Zanamivir pharmacokinetics and pulmonary penetration into epithelial lining fluid following intravenous or oral inhaled administration to healthy adult subjects. *Antimicrob. Agents Chemother.* **2011**, *55*, 5178–5184. https://doi.org/10.1128/aac.00703-11.
3. Ishizuka, H.; Toyama, K.; Yoshiba, S.; Okabe, H.; Furuie, H. Intrapulmonary distribution and pharmacokinetics of laninamivir, a neuraminidase inhibitor, after a single inhaled administration of its prodrug, laninamivir octanoate, in healthy volunteers. *Antimicrob. Agents Chemother.* **2012**, *56*, 3873–3878.
4. Toyama, K.; Furuie, H.; Ishizuka, H. Safety and Pharmacokinetics of Nebulized Laninamivir Octanoate, A Long Acting Neuraminidase Inhibitor, In Healthy Subjects. *Clin. Ther.* **2017**, *39*, e25–e26.
5. Englund, J.A.; Piedra, P.A.; Jefferson, L.S.; Wilson, S.Z.; Taber, L.H.; Gilbert, B.E. High-dose, short-duration ribavirin aerosol therapy in children with suspected respiratory syncytial virus infection. *J. Pediatr.* **1990**, *117*, 313–320.
6. Linn, W.S.; Gong, H.; Anderson, K.R.; Clark, K.W.; Shamoo, D.A. Exposures of Health-Care Workers to Ribavirin Aerosol: A Pharmacokinetic Study. *Arch. Environ. Health: Int. J.* **1995**, *50*, 445–451. https://doi.org/10.1080/00039896.1995.9935981.
7. Cass, L.M.R.; Efthymiopoulos, C.; Bye, A. Pharmacokinetics of zanamivir after intravenous, oral, inhaled or intranasal administration to healthy volunteers. *Clin. Pharmacokinet.* **1999**, *36*, 1–11.
8. Weller, S.; Jones, L.S.; Lou, Y.; Piscitelli, S.; Peppercorn, A.; Ng-Cashin, J. *Safety, Tolerability and Pharmacokinetics of Orally inhaled Zanamivir: A Randomized Study Comparing Rotacap/Rotahaler and Rotadisk/Diskhaler in Healthy Adults*; SAGE Publications Sage UK: London,UK, 2013.
9. Cass, L.M.R.; Brown, J.; Pickford, M.; Fayinka, S.; Newman, S.P.; Johansson, C.J.; Bye, A. Pharmacoscintigraphic evaluation of lung deposition of inhaled zanamivir in healthy volunteers. *Clin. Pharmacokinet.* **1999**, *36*, 21–31.
10. Peng, A.W.; Hussey, E.K.; Moore, K.H.P. A population pharmacokinetic analysis of zanamivir in subjects with experimental and naturally occurring influenza: Effects of formulation and route of administration. *J. Clin. Pharmacol.* **2000**, *40*, 242–249.
11. Ishizuka, H.; Yoshiba, S.; Yoshihara, K.; Okabe, H. Assessment of the effects of renal impairment on the pharmacokinetic profile of laninamivir, a novel neuraminidase inhibitor, after a single inhaled dose of its Prodrug, CS-8958. *J. Clin. Pharmacol.* **2011**, *51*, 243–251.
12. Ishizuka, H.; Yoshiba, S.; Okabe, H.; Yoshihara, K. Clinical pharmacokinetics of laninamivir, a novel long-acting neuraminidase inhibitor, after single and multiple inhaled doses of its prodrug, CS-8958, in healthy male volunteers. *J. Clin. Pharmacol.* **2010**, *50*, 1319–1329.
13. Yoshiba, S.; Okabe, H.; Ishizuka, H. Pharmacokinetics of laninamivir after a single administration of its prodrug, laninamivir octanoate, a long-acting neuraminidase inhibitor, using an easy-to-use inhaler in healthy volunteers. *J. Bioequiv. Availab.* **2011**, *3*, 001–004.
14. Yoshihara, K.; Ishizuka, H.; Kubo, Y. Population Pharmacokinetics of Laninamivir and Its Prodrug Laninamivir Octanoate in Healthy Subjects and in Adult and Pediatric Patients with Influenza Virus Infection. *Drug Metab. Pharmacokinet.* **2013**, *28*, 416–426. https://doi.org/10.2133/dmpk.DMPK-12-RG-115.
15. Couroux, P.; Brkovic, A.; Vittitow, J.L.; Israel, R.J.; Pamidi, C.; Patel, J.; Barakat, M. A randomized, placebo-controlled study to evaluate safety and pharmacokinetics of inhaled ribavirin. *Clin. Transl. Sci.* **2022**, *15*, 2159–2171.
16. Dumont, E.F.; Oliver, A.J.; Ioannou, C.; Billiard, J.; Dennison, J.; Van Den Berg, F.; Yang, S.; Chandrasekaran, V.; Young, G.C.; Lahiry, A. A novel inhaled dry-powder formulation of ribavirin allows for efficient lung delivery in healthy participants and those with chronic obstructive pulmonary disease in a phase 1 study. *Antimicrob. Agents Chemother.* **2020**, *64*, e02267-02219.
17. Atmar, R.L.; Greenberg, S.B.; Quarles, J.M.; Wilson, S.Z.; Tyler, B.; Feldman, S.; Couch, R.B. Safety and pharmacokinetics of rimantadine small-particle aerosol. *Antimicrob. Agents Chemother.* **1990**, *34*, 2228–2233.
